# Chloridocyclo­hexyl[(1,2,5,6-η)-cyclo­octa-1,5-diene]platinum(II)

**DOI:** 10.1107/S1600536809050910

**Published:** 2009-11-28

**Authors:** Kwang Ha

**Affiliations:** a School of Applied Chemical Engineering, The Research Institute of Catalysis, Chonnam National University, Gwangju 500-757, Republic of Korea

## Abstract

In the title complex, [Pt(C_6_H_11_)Cl(C_8_H_12_)], the Pt^II^ ion lies in a distorted square-planar environment defined by the Cl and cyclo­hexyl C atoms and the mid-points of the two π-coordinated double bonds of cyclo­octa-1,5-diene. As a result of the different *trans* influences of the Cl atom and the cyclo­hexyl group, the Pt—C bonds *trans* to the cyclo­hexyl group are longer than those *trans* to the Cl atom.

## Related literature

For the crystal structure of [(cod)PtCl_2_] (cod = cyclo­octa-1,5-diene), see: Goel *et al.* (1982 ▶); Syed *et al.* (1984 ▶). For the crystal structures of [(cod)Pt(CH_3_)*L*] (*L* = OH, CH_3_ or Cl), see: Klein *et al.* (1999 ▶).
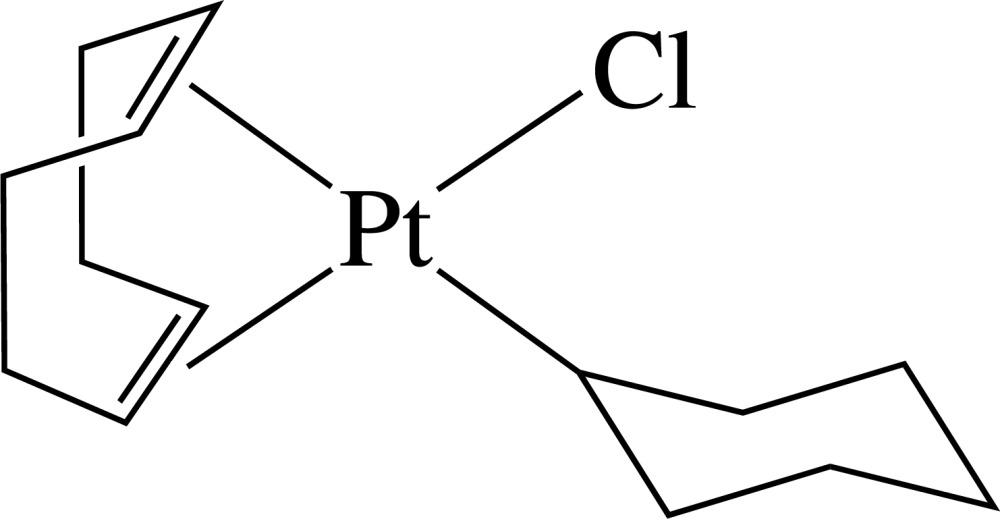


## Experimental

### 

#### Crystal data

[Pt(C_6_H_11_)Cl(C_8_H_12_)]
*M* *_r_* = 421.86
Monoclinic, 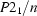

*a* = 10.6505 (5) Å
*b* = 12.3514 (6) Å
*c* = 11.1609 (6) Å
β = 105.175 (1)°
*V* = 1417.01 (12) Å^3^
*Z* = 4
Mo *K*α radiation
μ = 10.06 mm^−1^
*T* = 296 K
0.20 × 0.16 × 0.15 mm

#### Data collection

Bruker SMART 1000 CCD diffractometer
Absorption correction: multi-scan (*SADABS*; Bruker, 2000 ▶) *T*_min_ = 0.122, *T*_max_ = 0.221
10108 measured reflections
3481 independent reflections
2343 reflections with *I* > 2σ(*I*)
*R*_int_ = 0.050

#### Refinement

*R*[*F* ^2^ > 2σ(*F* ^2^)] = 0.034
*wR*(*F* ^2^) = 0.083
*S* = 1.12
3481 reflections
145 parameters
H-atom parameters constrained
Δρ_max_ = 1.12 e Å^−3^
Δρ_min_ = −1.79 e Å^−3^

### 

Data collection: *SMART* (Bruker, 2000 ▶); cell refinement: *SAINT* (Bruker, 2000 ▶); data reduction: *SAINT*; program(s) used to solve structure: *SHELXS97* (Sheldrick, 2008 ▶); program(s) used to refine structure: *SHELXL97* (Sheldrick, 2008 ▶); molecular graphics: *ORTEP-3* (Farrugia, 1997 ▶) and *PLATON* (Spek, 2009 ▶); software used to prepare material for publication: *SHELXL97*.

## Supplementary Material

Crystal structure: contains datablocks global, I. DOI: 10.1107/S1600536809050910/ng2696sup1.cif

Structure factors: contains datablocks I. DOI: 10.1107/S1600536809050910/ng2696Isup2.hkl

Additional supplementary materials:  crystallographic information; 3D view; checkCIF report

## Figures and Tables

**Fig. 1.**
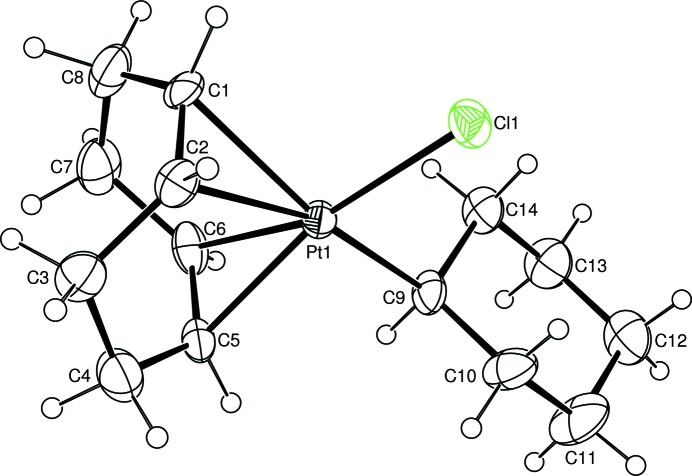
The structure of the title compound, with displacement ellipsoids drawn at the 30% probability level for non-H atoms.

**Fig. 2.**
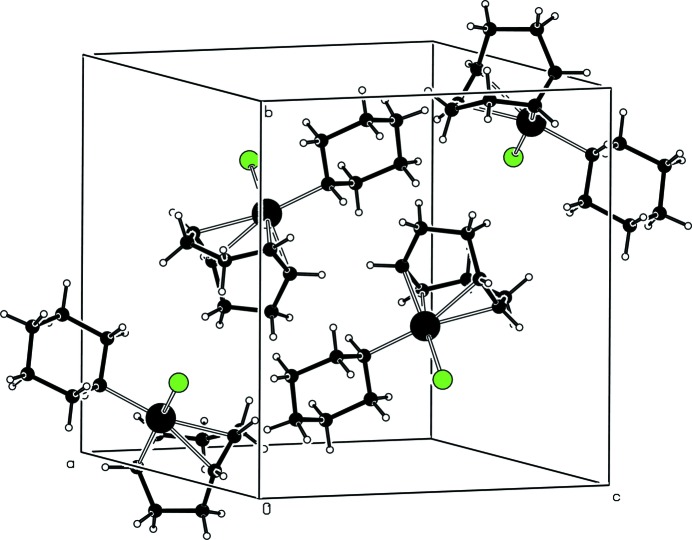
View of the unit-cell contents of the title compound.

## supplementary crystallographic information

### Comment

In the title complex, [Pt(C_6_H_11_)Cl(C_8_H_12_)], the central Pt^II^ ion lies in a distorted square-planar environment defined by the Cl and cyclohexyl C atoms and the two mid-points (M1, M2) of the π-coordinated double bonds of cycloocta-1,5-diene (cod) ligand (M1 and M2 denote the mid-points of the olefinic bonds C1—C2 and C5—C6, respectively) (Fig. 1 and Fig. 2). The Pt, Cl, C9 atoms and the mid-points lie in a coordination plane with the largest deviation of 0.021 Å (C9) from the least-squares plane, and with bond angles in the range of 85.2°–92.2°. Because of the different *trans* influences of the Cl atom and the cyclohexyl group, the Pt—C bonds *trans* to C9 of the cyclohexyl group are on average 0.215 Å longer than those *trans* to the Cl atom (Pt1—C1/C2 = 2.316 (7) and 2.337 (8) Å, Pt1—C5/C6 = 2.111 (8) and 2.112 (8) Å). The distances between the Pt atom and the mid-points are 2.228 Å (M1) and 1.992 Å (M2). The cod ligand coordinates to the Pt atom in the twist-boat conformation with the coordinated double-bond lengths of 1.339 (11) and 1.402 (12) Å, and the cod ring angles lie in the range of 113.0 (8)°–127.1 (8)°. The σ-bonded cyclohexyl ring is in the chair conformation with the ring angles of 109.9 (8)°–112.0 (8)°.

### Experimental

To a suspension of [(cod)PtCl_2_] (0.5015 g, 1.34 mmol) in ether (30 ml) was added cyclohexylmagnesium chloride (2.0 *M* solution in ether, 4.1 ml, 8.04 mmol) and stirred for 24 h at -5 °C. After methanolysis with 1 ml MeOH, the resulting mixture was diluted with ether (20 ml), and then filtered directly through a plug of Al_2_O_3_ (3 cm *x* 2 cm) and eluted with ether (80 ml). The solvent was removed under vacuum and the residue was dried, to give a white powder (0.3655 g). Crystals suitable for X-ray analysis were obtained by slow evaporation from an ethyl acetate solution.

### Refinement

H atoms were positioned geometrically and allowed to ride on their respective parent atoms [C—H = 0.98 (CH) or 0.97 (CH_2_) Å and *U*_iso_(H) = 1.2*U*_eq_(C)].

### Crystal data

**Table:**

[Pt(C_6_H_11_)Cl(C_8_H_12_)]	*F*(000) = 808
*M**_r_* = 421.86	*D*_x_ = 1.977 Mg m^−^^3^
Monoclinic, *P*2_1_/*n*	Mo *K*α radiation, λ = 0.71073 Å
Hall symbol: -P 2yn	Cell parameters from 3669 reflections
*a* = 10.6505 (5) Å	θ = 2.4–27.8°
*b* = 12.3514 (6) Å	µ = 10.06 mm^−^^1^
*c* = 11.1609 (6) Å	*T* = 296 K
β = 105.175 (1)°	Block, colorless
*V* = 1417.01 (12) Å^3^	0.20 × 0.16 × 0.15 mm
*Z* = 4	

### Data collection

**Table:**

Bruker SMART 1000 CCD diffractometer	3481 independent reflections
Radiation source: fine-focus sealed tube	2343 reflections with *I* > 2σ(*I*)
graphite	*R*_int_ = 0.050
φ and ω scans	θ_max_ = 28.3°, θ_min_ = 2.4°
Absorption correction: multi-scan (*SADABS*; Bruker, 2000)	*h* = −14→14
*T*_min_ = 0.122, *T*_max_ = 0.221	*k* = −8→16
10108 measured reflections	*l* = −14→14

### Refinement

**Table:**

Refinement on *F*^2^	Primary atom site location: structure-invariant direct methods
Least-squares matrix: full	Secondary atom site location: difference Fourier map
*R*[*F*^2^ > 2σ(*F*^2^)] = 0.034	Hydrogen site location: inferred from neighbouring sites
*wR*(*F*^2^) = 0.083	H-atom parameters constrained
*S* = 1.12	*w* = 1/[σ^2^(*F*_o_^2^) + (0.0112*P*)^2^ + 4.7457*P*] where *P* = (*F*_o_^2^ + 2*F*_c_^2^)/3
3481 reflections	(Δ/σ)_max_ < 0.001
145 parameters	Δρ_max_ = 1.12 e Å^−^^3^
0 restraints	Δρ_min_ = −1.79 e Å^−^^3^

### Special details

**Table:**

Geometry. All e.s.d.'s (except the e.s.d. in the dihedral angle between two l.s. planes) are estimated using the full covariance matrix. The cell e.s.d.'s are taken into account individually in the estimation of e.s.d.'s in distances, angles and torsion angles; correlations between e.s.d.'s in cell parameters are only used when they are defined by crystal symmetry. An approximate (isotropic) treatment of cell e.s.d.'s is used for estimating e.s.d.'s involving l.s. planes.
Refinement. Refinement of *F*^2^ against ALL reflections. The weighted *R*-factor *wR* and goodness of fit *S* are based on *F*^2^, conventional *R*-factors *R* are based on *F*, with *F* set to zero for negative *F*^2^. The threshold expression of *F*^2^ > σ(*F*^2^) is used only for calculating *R*-factors(gt) *etc*. and is not relevant to the choice of reflections for refinement. *R*-factors based on *F*^2^ are statistically about twice as large as those based on *F*, and *R*- factors based on ALL data will be even larger.

### Fractional atomic coordinates and isotropic or equivalent isotropic displacement parameters (Å^2^)

**Table:**

	*x*	*y*	*z*	*U*_iso_*/*U*_eq_	
Pt1	0.23435 (3)	0.38659 (3)	0.59178 (3)	0.03388 (11)	
Cl1	0.0531 (2)	0.2747 (2)	0.5501 (2)	0.0491 (6)	
C1	0.1992 (9)	0.4337 (7)	0.7812 (7)	0.043 (2)	
H1	0.1372	0.3870	0.8078	0.051*	
C2	0.1440 (8)	0.5121 (8)	0.7019 (8)	0.044 (2)	
H2	0.0487	0.5132	0.6801	0.052*	
C3	0.2067 (9)	0.6204 (8)	0.6984 (8)	0.053 (2)	
H3A	0.2625	0.6364	0.7802	0.064*	
H3B	0.1396	0.6755	0.6785	0.064*	
C4	0.2864 (10)	0.6255 (8)	0.6048 (9)	0.063 (3)	
H4A	0.2299	0.6477	0.5256	0.076*	
H4B	0.3522	0.6812	0.6308	0.076*	
C5	0.3523 (8)	0.5229 (7)	0.5861 (8)	0.043 (2)	
H5	0.3865	0.5243	0.5128	0.052*	
C6	0.4195 (8)	0.4513 (8)	0.6782 (9)	0.052 (3)	
H6	0.4921	0.4135	0.6578	0.062*	
C7	0.4382 (10)	0.4719 (9)	0.8158 (9)	0.068 (3)	
H7A	0.5169	0.4351	0.8607	0.082*	
H7B	0.4522	0.5489	0.8307	0.082*	
C8	0.3306 (11)	0.4376 (9)	0.8696 (8)	0.065 (3)	
H8A	0.3276	0.4870	0.9364	0.078*	
H8B	0.3510	0.3663	0.9058	0.078*	
C9	0.3060 (8)	0.3085 (8)	0.4561 (8)	0.044 (2)	
H9	0.3882	0.3438	0.4554	0.053*	
C10	0.2166 (11)	0.3210 (9)	0.3290 (8)	0.064 (3)	
H10A	0.2023	0.3974	0.3099	0.076*	
H10B	0.1332	0.2883	0.3267	0.076*	
C11	0.2741 (13)	0.2672 (11)	0.2310 (10)	0.087 (4)	
H11A	0.2116	0.2718	0.1504	0.105*	
H11B	0.3517	0.3060	0.2262	0.105*	
C12	0.3075 (11)	0.1516 (9)	0.2608 (10)	0.071 (3)	
H12A	0.3513	0.1227	0.2017	0.085*	
H12B	0.2282	0.1105	0.2530	0.085*	
C13	0.3945 (10)	0.1387 (9)	0.3909 (9)	0.067 (3)	
H13A	0.4785	0.1713	0.3952	0.080*	
H13B	0.4083	0.0622	0.4096	0.080*	
C14	0.3359 (9)	0.1916 (8)	0.4883 (8)	0.051 (2)	
H14A	0.2567	0.1540	0.4911	0.061*	
H14B	0.3968	0.1863	0.5696	0.061*	

### Atomic displacement parameters (Å^2^)

**Table:**

	*U*^11^	*U*^22^	*U*^33^	*U*^12^	*U*^13^	*U*^23^
Pt1	0.03384 (17)	0.03547 (19)	0.03338 (16)	−0.00364 (17)	0.01065 (11)	−0.00381 (18)
Cl1	0.0403 (12)	0.0527 (15)	0.0538 (13)	−0.0132 (10)	0.0116 (10)	−0.0063 (12)
C1	0.070 (6)	0.037 (5)	0.028 (4)	0.006 (5)	0.025 (4)	−0.004 (4)
C2	0.042 (5)	0.048 (6)	0.043 (5)	0.007 (4)	0.015 (4)	−0.006 (5)
C3	0.056 (6)	0.054 (7)	0.048 (5)	0.012 (5)	0.010 (4)	0.002 (5)
C4	0.075 (7)	0.046 (7)	0.072 (7)	−0.006 (5)	0.026 (6)	−0.009 (6)
C5	0.049 (5)	0.044 (6)	0.049 (5)	−0.020 (4)	0.031 (4)	−0.019 (5)
C6	0.030 (5)	0.053 (6)	0.068 (6)	−0.015 (4)	0.005 (4)	−0.018 (6)
C7	0.062 (7)	0.067 (8)	0.058 (6)	0.000 (6)	−0.014 (5)	−0.007 (6)
C8	0.096 (9)	0.055 (7)	0.035 (5)	0.017 (6)	0.000 (5)	−0.006 (5)
C9	0.049 (5)	0.047 (6)	0.042 (5)	−0.008 (4)	0.022 (4)	−0.018 (4)
C10	0.096 (8)	0.056 (7)	0.046 (6)	0.018 (6)	0.033 (6)	0.007 (5)
C11	0.121 (11)	0.108 (12)	0.047 (6)	0.015 (9)	0.046 (7)	−0.009 (7)
C12	0.084 (8)	0.061 (8)	0.080 (8)	0.000 (6)	0.042 (7)	−0.024 (7)
C13	0.072 (7)	0.064 (8)	0.072 (7)	0.010 (6)	0.032 (6)	−0.012 (6)
C14	0.056 (6)	0.045 (6)	0.057 (6)	0.008 (5)	0.026 (5)	−0.013 (5)

### Geometric parameters (Å, °)

**Table:**

Pt1—C9	2.100 (8)	C7—H7A	0.9700
Pt1—C5	2.111 (8)	C7—H7B	0.9700
Pt1—C6	2.112 (8)	C8—H8A	0.9700
Pt1—C1	2.316 (7)	C8—H8B	0.9700
Pt1—Cl1	2.320 (2)	C9—C10	1.495 (12)
Pt1—C2	2.337 (8)	C9—C14	1.502 (12)
C1—C2	1.339 (11)	C9—H9	0.9800
C1—C8	1.487 (13)	C10—C11	1.538 (12)
C1—H1	0.9800	C10—H10A	0.9700
C2—C3	1.500 (13)	C10—H10B	0.9700
C2—H2	0.9800	C11—C12	1.488 (15)
C3—C4	1.510 (12)	C11—H11A	0.9700
C3—H3A	0.9700	C11—H11B	0.9700
C3—H3B	0.9700	C12—C13	1.513 (14)
C4—C5	1.490 (13)	C12—H12A	0.9700
C4—H4A	0.9700	C12—H12B	0.9700
C4—H4B	0.9700	C13—C14	1.534 (11)
C5—C6	1.402 (12)	C13—H13A	0.9700
C5—H5	0.9800	C13—H13B	0.9700
C6—C7	1.517 (12)	C14—H14A	0.9700
C6—H6	0.9800	C14—H14B	0.9700
C7—C8	1.487 (14)		
			
C9—Pt1—C5	90.8 (3)	Pt1—C6—H6	114.4
C9—Pt1—C6	91.8 (4)	C8—C7—C6	116.8 (8)
C5—Pt1—C6	38.8 (3)	C8—C7—H7A	108.1
C9—Pt1—C1	161.8 (3)	C6—C7—H7A	108.1
C5—Pt1—C1	93.8 (3)	C8—C7—H7B	108.1
C6—Pt1—C1	80.9 (4)	C6—C7—H7B	108.1
C9—Pt1—Cl1	91.3 (2)	H7A—C7—H7B	107.3
C5—Pt1—Cl1	159.9 (3)	C7—C8—C1	115.6 (8)
C6—Pt1—Cl1	160.9 (3)	C7—C8—H8A	108.4
C1—Pt1—Cl1	90.5 (2)	C1—C8—H8A	108.4
C9—Pt1—C2	164.0 (3)	C7—C8—H8B	108.4
C5—Pt1—C2	79.5 (3)	C1—C8—H8B	108.4
C6—Pt1—C2	88.4 (4)	H8A—C8—H8B	107.4
C1—Pt1—C2	33.5 (3)	C10—C9—C14	111.7 (8)
Cl1—Pt1—C2	93.7 (2)	C10—C9—Pt1	112.0 (6)
C2—C1—C8	126.1 (9)	C14—C9—Pt1	111.3 (6)
C2—C1—Pt1	74.1 (5)	C10—C9—H9	107.2
C8—C1—Pt1	105.4 (6)	C14—C9—H9	107.2
C2—C1—H1	114.3	Pt1—C9—H9	107.2
C8—C1—H1	114.3	C9—C10—C11	110.9 (9)
Pt1—C1—H1	114.3	C9—C10—H10A	109.5
C1—C2—C3	122.7 (8)	C11—C10—H10A	109.5
C1—C2—Pt1	72.4 (5)	C9—C10—H10B	109.5
C3—C2—Pt1	109.3 (6)	C11—C10—H10B	109.5
C1—C2—H2	114.9	H10A—C10—H10B	108.0
C3—C2—H2	114.9	C12—C11—C10	111.9 (9)
Pt1—C2—H2	114.9	C12—C11—H11A	109.2
C2—C3—C4	113.0 (8)	C10—C11—H11A	109.2
C2—C3—H3A	109.0	C12—C11—H11B	109.2
C4—C3—H3A	109.0	C10—C11—H11B	109.2
C2—C3—H3B	109.0	H11A—C11—H11B	107.9
C4—C3—H3B	109.0	C11—C12—C13	111.5 (10)
H3A—C3—H3B	107.8	C11—C12—H12A	109.3
C5—C4—C3	115.5 (9)	C13—C12—H12A	109.3
C5—C4—H4A	108.4	C11—C12—H12B	109.3
C3—C4—H4A	108.4	C13—C12—H12B	109.3
C5—C4—H4B	108.4	H12A—C12—H12B	108.0
C3—C4—H4B	108.4	C12—C13—C14	112.0 (8)
H4A—C4—H4B	107.5	C12—C13—H13A	109.2
C6—C5—C4	127.1 (8)	C14—C13—H13A	109.2
C6—C5—Pt1	70.7 (5)	C12—C13—H13B	109.2
C4—C5—Pt1	111.6 (6)	C14—C13—H13B	109.2
C6—C5—H5	113.2	H13A—C13—H13B	107.9
C4—C5—H5	113.2	C9—C14—C13	109.9 (8)
Pt1—C5—H5	113.2	C9—C14—H14A	109.7
C5—C6—C7	122.9 (9)	C13—C14—H14A	109.7
C5—C6—Pt1	70.6 (5)	C9—C14—H14B	109.7
C7—C6—Pt1	112.7 (6)	C13—C14—H14B	109.7
C5—C6—H6	114.4	H14A—C14—H14B	108.2
C7—C6—H6	114.4		
			
C9—Pt1—C1—C2	168.5 (9)	C9—Pt1—C6—C5	89.0 (6)
C5—Pt1—C1—C2	64.3 (6)	C1—Pt1—C6—C5	−107.8 (5)
C6—Pt1—C1—C2	101.0 (6)	Cl1—Pt1—C6—C5	−171.9 (6)
Cl1—Pt1—C1—C2	−96.1 (6)	C2—Pt1—C6—C5	−75.0 (5)
C9—Pt1—C1—C8	44.6 (14)	C9—Pt1—C6—C7	−152.5 (8)
C5—Pt1—C1—C8	−59.5 (7)	C5—Pt1—C6—C7	118.5 (10)
C6—Pt1—C1—C8	−22.8 (7)	C1—Pt1—C6—C7	10.6 (7)
Cl1—Pt1—C1—C8	140.1 (6)	Cl1—Pt1—C6—C7	−53.4 (13)
C2—Pt1—C1—C8	−123.8 (9)	C2—Pt1—C6—C7	43.4 (8)
C8—C1—C2—C3	−4.3 (14)	C5—C6—C7—C8	84.8 (12)
Pt1—C1—C2—C3	−102.0 (8)	Pt1—C6—C7—C8	4.0 (12)
C8—C1—C2—Pt1	97.7 (9)	C6—C7—C8—C1	−26.2 (14)
C9—Pt1—C2—C1	−166.9 (10)	C2—C1—C8—C7	−49.3 (13)
C5—Pt1—C2—C1	−113.8 (6)	Pt1—C1—C8—C7	32.0 (10)
C6—Pt1—C2—C1	−75.9 (6)	C5—Pt1—C9—C10	−97.5 (7)
Cl1—Pt1—C2—C1	85.2 (6)	C6—Pt1—C9—C10	−136.2 (7)
C9—Pt1—C2—C3	−47.6 (15)	C1—Pt1—C9—C10	157.9 (9)
C5—Pt1—C2—C3	5.4 (6)	Cl1—Pt1—C9—C10	62.6 (7)
C6—Pt1—C2—C3	43.4 (6)	C2—Pt1—C9—C10	−45.7 (15)
C1—Pt1—C2—C3	119.3 (9)	C5—Pt1—C9—C14	136.7 (7)
Cl1—Pt1—C2—C3	−155.5 (6)	C6—Pt1—C9—C14	98.0 (7)
C1—C2—C3—C4	93.5 (10)	C1—Pt1—C9—C14	32.1 (14)
Pt1—C2—C3—C4	12.5 (9)	Cl1—Pt1—C9—C14	−63.2 (6)
C2—C3—C4—C5	−33.3 (12)	C2—Pt1—C9—C14	−171.5 (10)
C3—C4—C5—C6	−43.5 (13)	C14—C9—C10—C11	−56.8 (11)
C3—C4—C5—Pt1	38.1 (10)	Pt1—C9—C10—C11	177.6 (7)
C9—Pt1—C5—C6	−91.9 (6)	C9—C10—C11—C12	54.8 (14)
C1—Pt1—C5—C6	70.4 (6)	C10—C11—C12—C13	−53.3 (14)
Cl1—Pt1—C5—C6	172.3 (6)	C11—C12—C13—C14	54.1 (13)
C2—Pt1—C5—C6	100.8 (6)	C10—C9—C14—C13	56.9 (10)
C9—Pt1—C5—C4	144.8 (7)	Pt1—C9—C14—C13	−177.1 (6)
C6—Pt1—C5—C4	−123.2 (9)	C12—C13—C14—C9	−55.3 (12)
C1—Pt1—C5—C4	−52.8 (7)	C1—C2—C5—C6	−7.2 (7)
Cl1—Pt1—C5—C4	49.1 (11)	C3—C4—C7—C8	22.4 (8)
C2—Pt1—C5—C4	−22.5 (7)	C3—C2—C1—C8	−4.3 (14)
C4—C5—C6—C7	−2.0 (14)	C4—C5—C6—C7	−2.0 (14)
Pt1—C5—C6—C7	−105.0 (9)	C2—C3—C4—C5	−33.3 (12)
C4—C5—C6—Pt1	103.0 (9)	C6—C7—C8—C1	−26.2 (14)
